# Gastric cancer with large bowel obstruction as the first presentation: A case report

**DOI:** 10.3892/ol.2013.1540

**Published:** 2013-08-22

**Authors:** XIUYAN YU, JUNSHU ZHANG

**Affiliations:** 1 Department of Surgery, Second Affiliated Hospital (Binjiang Branch), Zhejiang University School of Medicine, Hangzhou, Zhejiang 310009, P.R. China; 2 Department of Surgery, Linan People’s Hospital, Hangzhou, Zhejiang 311300, P.R. China

**Keywords:** gastric cancer, colon, invasion, obstruction

## Abstract

Gastric adenocarcinoma with a large bowel obstruction as the first presentation of the condition is rare. The present study describes the case of a 59-year-old female who was diagnosed with a large bowel obstruction that was caused by gastric adenocarcinoma. The patient suffered from abdominal pain and had not defecated for 15 days. The patient had no significant medical history. The right epigastric region was slightly tender and active bowel sounds were identified. A computed tomography (CT) scan revealed a dilated fluid-filled colon with a thickened wall and adjacent fat infiltration. An upper gastrointestinal endoscopy revealed that the margin and gastric mucosa in the antrum were raised and thickened without evidence of ulcerative lesions. The patient underwent surgery and was administered adjuvant chemotherapy. The patient was followed-up for 18 months without recurrence of the tumor. This study demonstrates that the presentation of gastric cancer may vary.

## Introduction

Gastric cancer is the second leading cause of cancer-related mortality worldwide (1,2). The various clinical manifestations in certain patients make the diagnosis difficult and indicate a poor prognosis. Although gastrointestinal obstructions are not uncommon in gastric cancer (3–5), observing a large bowel obstruction as the first manifestation of the disease is rare. The present study describes a case of gastric cancer in a patient who presented with abdominal pain and an acute large bowel obstruction. To the best of our knowledge, the present study is the first in the literature to describe an initial manifestation of a large bowel obstruction associated with gastric cancer. Written informed consent was obtained from the patient.

## Case report

**Figure 1:**
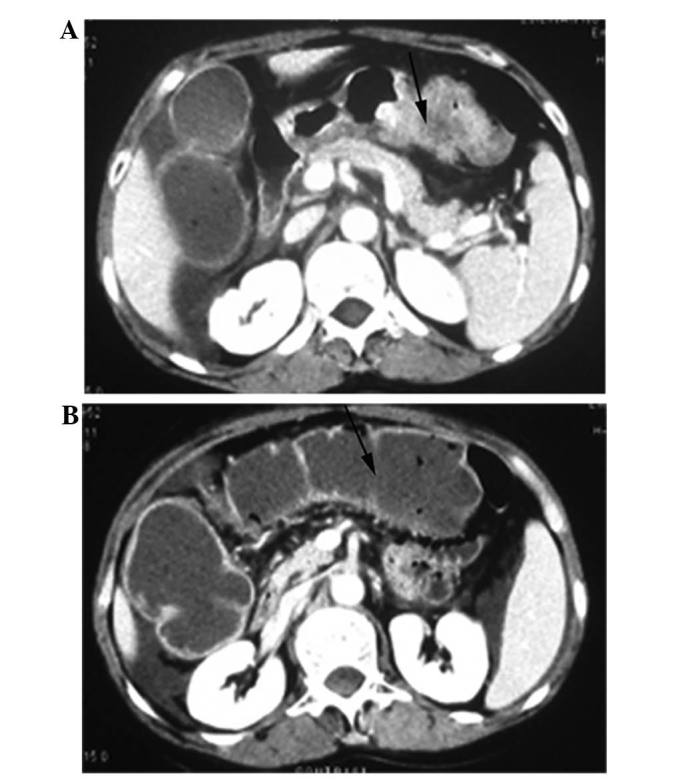
Computed tomography (CT) scan showing the gastric wall thickening and tumor infiltration into the (A) adjacent fat tissues and (B) colon, which were fluid-filled, indicative of a large bowel obstruction.

**Figure 2:**
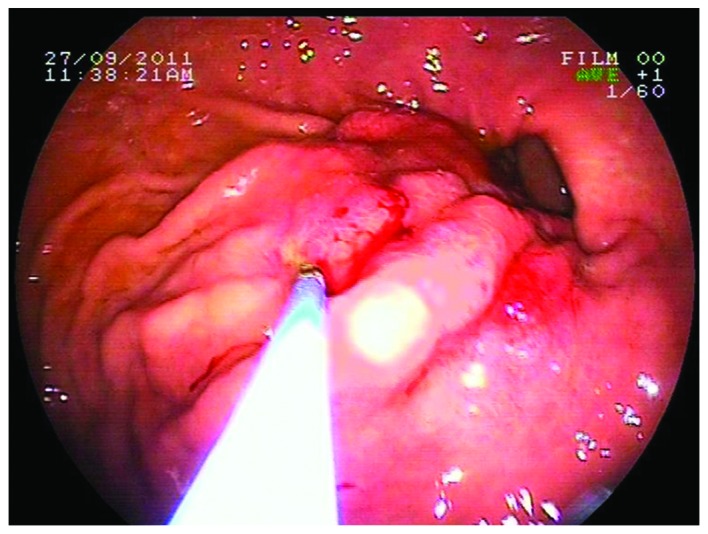
Gastroscopy demonstrating the margin and gastric mucosal in the antrum, which was raised and thickened. However, the mucosa did not contain ulcerative lesions.

A 59-year-old female without no significant medical history visited Linan People’s Hospital (Hangzhou, China) complaining of central abdominal pain and not being able to defecate for 15 days. Although the patient had been admitted to Hangzhou Provincial Hospital due to recurrent abdominal pain six months previously, the colon was not considered abnormal following a colonoscopy and ultrasound. A gastroscopy only revealed chronic superficial gastritis (data not shown). Upon admittance to the Department of Surgery, the right epigastric region was slightly tender and active bowel sounds were identified, followed by intermittent bilious vomiting and weight loss, despite a distended abdomen without rebound pain or hepatomegaly. Computed tomography (CT) of the abdomen revealed a dilated fluid-filled colon and a protruding tumor abutting the transverse colon. No other abnormalities/metastases to the liver or the involvement of adjacent organs/tissues were observed (Fig. 1). An upper gastrointestinal endoscopy revealed the existence of a raised margin and thickening of the gastric mucosal in the antrum, with no evidence of ulcerative lesions (Fig. 2). The endoscopic gastric biopsies indicated a diagnosis of a poorly-differentiated adenocarcinoma. The routine blood test results were normal, with the exception of an elevated CA19-9 level (95 U/l; normal range, <39 U/l).

**Figure 3:**
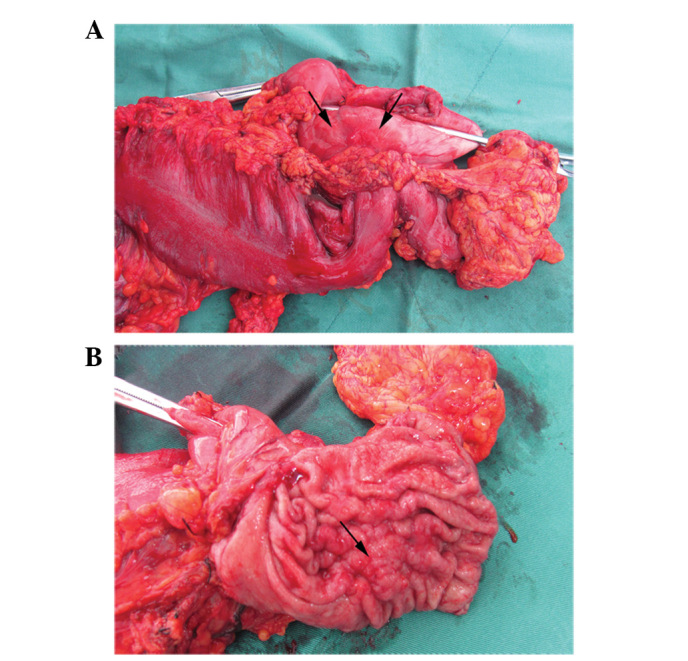
Post-operative surgical specimen of the tumor and adjacent tissues. (A) Transverse colon; (B) stomach.

**Figure 4:**
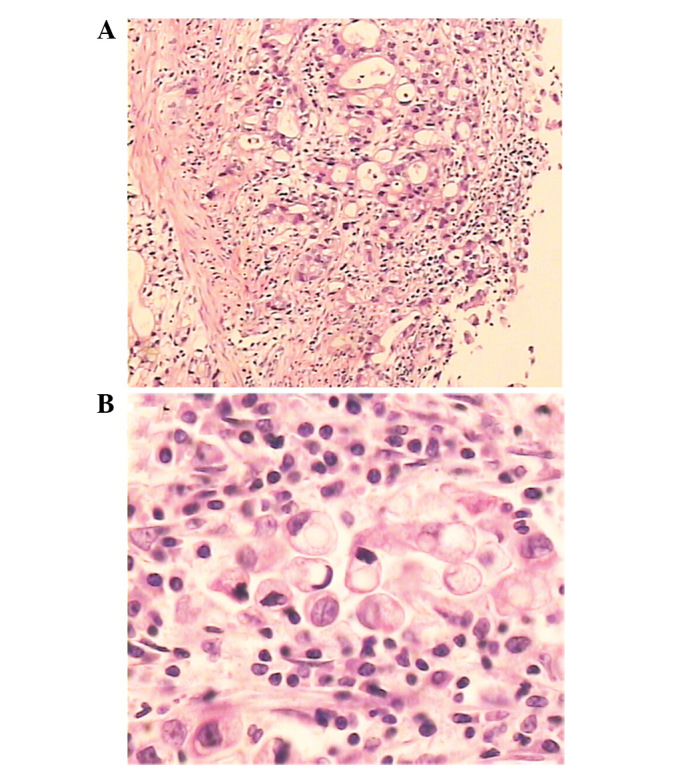
Microscopic appearance of the tumor (hematoxylin and eosin staining). Magnification, (A) ×10 and (B) ×40.

The patient underwent a radical distal gastrectomy with systematic lymph node dissection and a right colectomy (Fig. 3). The histology of the specimens indicated a poorly-differentiated adenocarcinoma with the presence of signet cells and cancer invasion to the transverse colonic mucosa (pT4; Fig. 4). No perigastric lymph node metastases were identified (0/15, pN0). The patient is currently doing well without recurrence at 18 months post-surgery.

## Discussion

Although highly advanced stage gastric cancer often causes gastric outlet obstruction and results in gastrointestinal symptoms, including abdominal pain, nausea and vomiting, an acute large bowel obstruction as the first presentation of gastric cancer is a rare event. The main route of cancer infiltrating the abdominal organs is known as the diffusion of the adjacent tissues and the invasion of the superficial serosal layer (6). In the present study, the wall of the transverse colon was thickened on the CT and a poorly-differentiated adenocarcinoma was confirmed by the histology examination, revealing the true invasion from the primary gastric cancer. Gastroscopy indicated that the gastric mucosa was partly raised and the distant organs did not contain any lesions. However, the cancer cells invaded the adjacent tissues, suggesting that the biological behavior of cancer cells and the performance and progress of gastric cancer may vary.

The poor prognosis of gastric cancer is mainly due to a late diagnosis (7). Therefore, an early diagnosis of the disease is the most significant factor in order to improve the results of treatment. However, an early diagnosis is difficult if a lesion is not identified in the stomach by a gastroscopy examination in the early stages. In certain cases, the diagnosis also relies on a combination of CT, magnifying endoscopy or endoscopic ultrasound techniques to analyze the symptoms, which are now the most common methods for detecting gastrointestinal invasion from gastric adenocarcinoma (8–11). The disease often characteristically manifests with a wall thickening detected by a helical CT. In the present case, the patient experienced abdomen pain, which may be associated with the oppression of the gastrointestinal tract due to cancer. The present study serves to illustrate the significance of a careful examination in all patients who present with symptoms of abdominal pain.
